# Acute left main coronary artery occlusion

**DOI:** 10.12669/pjms.291.2819

**Published:** 2013

**Authors:** K. Mehmet Burgazli, Mehmet Bilgin, Nedim Soydan, Ridvan Chasan, Ali Erdogan

**Affiliations:** 1 K. Mehmet Burgazli, Wuppertal Research and Medical Center, Department of Innere Medizin, Angiology Wuppertal, Germany.; 2 Mehmet Bilgin, Department of Radiology, Bezmialem Vakif University, Istanbul, Turkey.; 3 Nedim Soydan, University Clinic of Giessen, Internal Medicine, Cardiology, Angiology, Giessen, Germany.; 4 Ridvan Chasan , University Clinic of Giessen, Internal Medicine, Cardiology, Angiology, Giessen, Germany.; 5 Ali Erdogan, University Clinic of Giessen, Internal Medicine, Cardiology, Angiology, Giessen, Germany.

**Keywords:** Acute left main occlusion, Percutaneous coronary angioplasty, Bypass surgery

## Abstract

The treatment of an acute left main coronary artery occlusion still poses a challenge. In this case report we present a 50-year-old patient with an acute occlusion of the left main artery. After a successful angioplasty without “stenting” due to the complexity of the stenosis the patient underwent a successful bypass surgery. We discuss the therapeutic options of acute left main occlusion regarding medical, interventional and surgical options.

## Introduction

 Acute left main coronary artery occlusion presents a high-risk situation. The medical treatment of the unprotected left main coronary artery has high mortality.^1^ Besides the bypass surgery with an evident benefit in comparison with medical therapy, the percutaneous transluminal coronary angioplasty (PTCA) offers another therapeutic option.^2^

## Case Report

**Fig.1:**
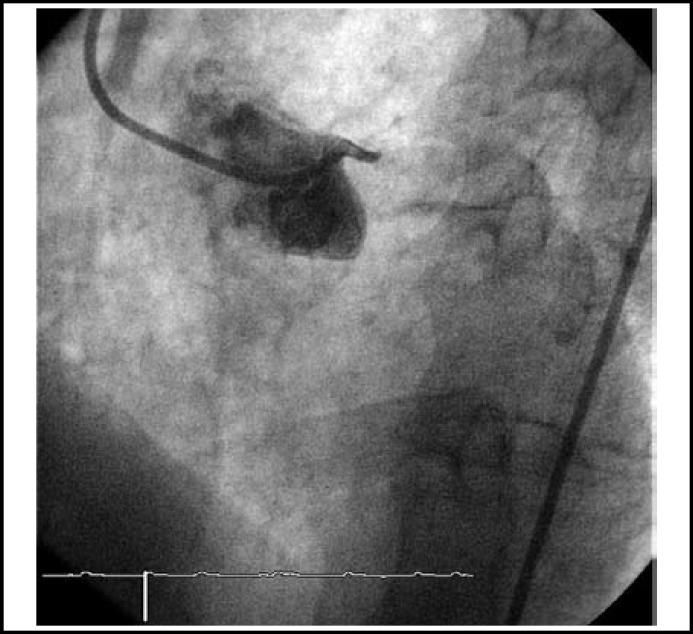
Occlusion of the left main

**Fig.2:**
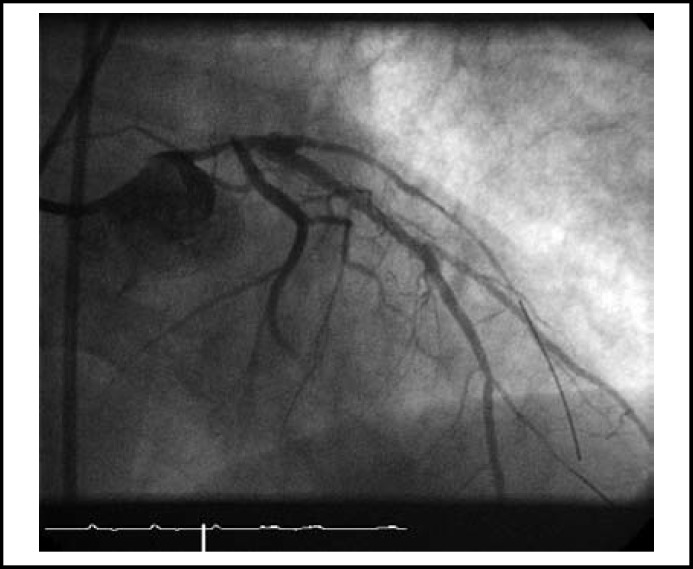
After single ballon dilatation of the left main

**Fig.3:**
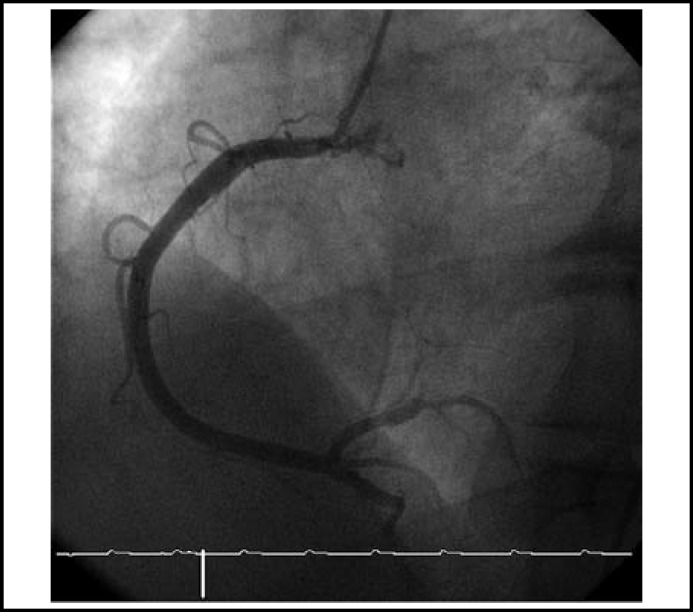
Right coronary artery

 A 50-year old male patient was admitted to our hospital in cardiogenic shock with ST-elevation myocardial infarction (STEMI). The cardiovascular risk factors were dyslipidemia, hypertension, and smoking. The coronary angiography (Fig. 1-3) presented a two vessel disease with a high-grade left main coronary artery occlusion {100% de-novo stenosis in the left main}. After successful PTCA the stenosis was reduced to 70%. The patient was supported with an intra- aortic balloon counterpulsation (IABP) device during phase of cardiac decompensation with acute pulmonary edema. He received low dose of catecholamines. The echocardiography documented a severely reduced left ventricular ejection fraction. After stabilization in our intensive care unit, the patient was then transferred to cardiac surgery. After coronary artery bypass grafting (CABG; left Arteria mammaria to LAD, Vena saphena magna to Ramus intermedius and to Ramus circumflexus) patient died the 11^th ^postoperative day due to septic shock.

## Discussion

 Acute occlusion of the left main coronary artery is rare and generally fatal. The mechanism is mostly acute thrombosis and the clinical presentation shows an extensive infarction usually with cardiogenic shock. Bypass surgery shows a survival benefit compared to medical therapy, and is the current standard of therapy for patients with unprotected left main coronary artery occlusion.^3^ The initial attempt of the percutaneous coronary intervention (PCI) documented frequent serious comorbidities and high event rates. In contrast the additional stenting of the unprotected left main coronary artery results in better prognosis. Since the appearance of drug eluting stents (DES) with reduction in restenosis rates, recent studies compared PCI with DES versus bypass surgery. In this connection PCI shows non-significant results with lower 6-month^4^ and 12-month mortality and myocardial infarction rates.^5^

 Nevertheless, there also exists data with significantly higher 6-month^4^ and 12-month target vessel revascularization rates,^5^ due to instent restenosis. To our knowledge, our case report represents one of the first time a patient with an acute left main coronary artery occlusion within STEMI, who received a successful PCI for the myocardial revascularization. In this case the PCI is used as a bridge over to CABG. The fact that the patient died the 11^th^ postoperative day due to non cardiac death, signifies basically the acute resolution. Patients with renal dysfunction, prior bypass surgery, advanced age, and severe heart failure are at highest risk. As mentioned our patient presented a highly reduced left ventricular ejection fraction. We may advise PCI as an alternative for patients who are ineligible for bypass surgery, or for emergency myocardial infarction as a bridge over to CABG in selected patients with acute left main occlusion like in our case report.
